# Optical Coherence Tomography in Acute Coronary Syndromes

**DOI:** 10.31083/RCM43321

**Published:** 2025-11-27

**Authors:** Andreas Synetos, Leonidas Koliastasis, Nikolaos Ktenopoulos, Svetlana Aghayan, Odysseas Katsaros, Konstantina Vlasopoulou, Maria Drakopoulou, Anastasios Apostolos, Ioannis Kachrimanidis, Panayotis K. Vlachakis, Elias Tolis, George Latsios, Konstantinos Tsioufis, Konstantinos Toutouzas

**Affiliations:** ^1^First Department of Cardiology, School of Medicine, National and Kapodistrian University of Athens, Hippokration General Hospital of Athens, 11527 Athens, Greece; ^2^Faculty of Medicine, European University of Cyprus, 2404 Egkomi, Cyprus; ^3^Cardiology Department, Lefkos Stavros Hospital, 11528 Athens, Greece

**Keywords:** ACS, acute coronary syndrome, intravascular imaging, OCT

## Abstract

Angiography remains the standard imaging modality during cardiac catheterization; however, this technique provides only a two-dimensional representation of the coronary lumen, which limits the assessment of vessel wall pathology. In comparison, intravascular imaging techniques, such as intravascular ultrasound (IVUS) and optical coherence tomography (OCT), provide high-resolution cross-sectional and two-dimensional reconstructions of the coronary arteries. Thus, these modalities complement angiographic findings, enable detailed evaluation of underlying pathology, and facilitate precise procedural guidance. Advancements in imaging technologies, including near-infrared spectroscopy and virtual histology intravascular ultrasound, further enhance lesion characterization and procedural planning. An increasing body of evidence from registries, randomized controlled trials, and meta-analyses supports the use of intravascular imaging-guided percutaneous coronary interventions, demonstrating improved procedural success rates and superior long-term clinical outcomes. In the context of acute coronary syndromes (ACS), OCT offers critical diagnostic insights that enhance accuracy and inform optimal treatment strategies. This review highlights the evolving role of OCT in the management of ACS and the favorable impact of this technique on patient outcomes.

## 1. Introduction

Angiography remains the primary imaging technique employed in the cardiac catheterization laboratory; however, it is subject to well-recognized limitations. These limitations stem from angiography generating a two-dimensional representation of the coronary lumen rather than visualizing the vessel wall, where atherosclerotic disease originates and progresses. The acute coronary syndromes (ACS) are a spectrum of pathophysiologic processes that result in partial or complete occlusion of the coronary vessel; with thrombus presence being their hallmark. To distinguish the patterns of ACS and guide our further treatment, intravascular imaging is required [1]. They include intravascular ultrasound (IVUS) and optical coherence tomography (OCT), offering cross-sectional tomographic views of the coronary arteries and providing insights that complement those obtained through angiography. Novel technologies as near-infrared spectroscopy and virtual histology ultrasound, expand our quiver in a quest to characterize and quantify the coronary lesions as well as optimize our interventions [2]. Evidence from registries, randomized controlled trials, and meta-analyses consistently demonstrates both procedural and long-term advantages of intravascular imaging-guided percutaneous coronary interventions (PCI) [3, 4]. OCT offers unique advantages in identifying thrombus and characterizing the intima and the underlying pathology. We aimed to provide a comprehensive review of the role of OCT in ACS.

## 2. OCT Basics

The first intravascular OCT system to become commercially available employed time-domain detection technology (TD-OCT). This system featured a catheter with an outer diameter of 0.019 inches, incorporating a 0.006-inch optical fiber, and was designed to resemble a guidewire (LightLab Imaging Inc., Westford, MA, USA). TD-OCT was limited by a relatively slow image acquisition rate of 15 to 20 frames per second (comprising 200–240 axial scans per frame), which constrained the maximum pullback speed to 2 mm per second. Consequently, imaging required proximal occlusion of coronary blood flow using a dedicated balloon, along with saline infusion delivered through the tip of the occlusion device [5].

**Fig. 1.**
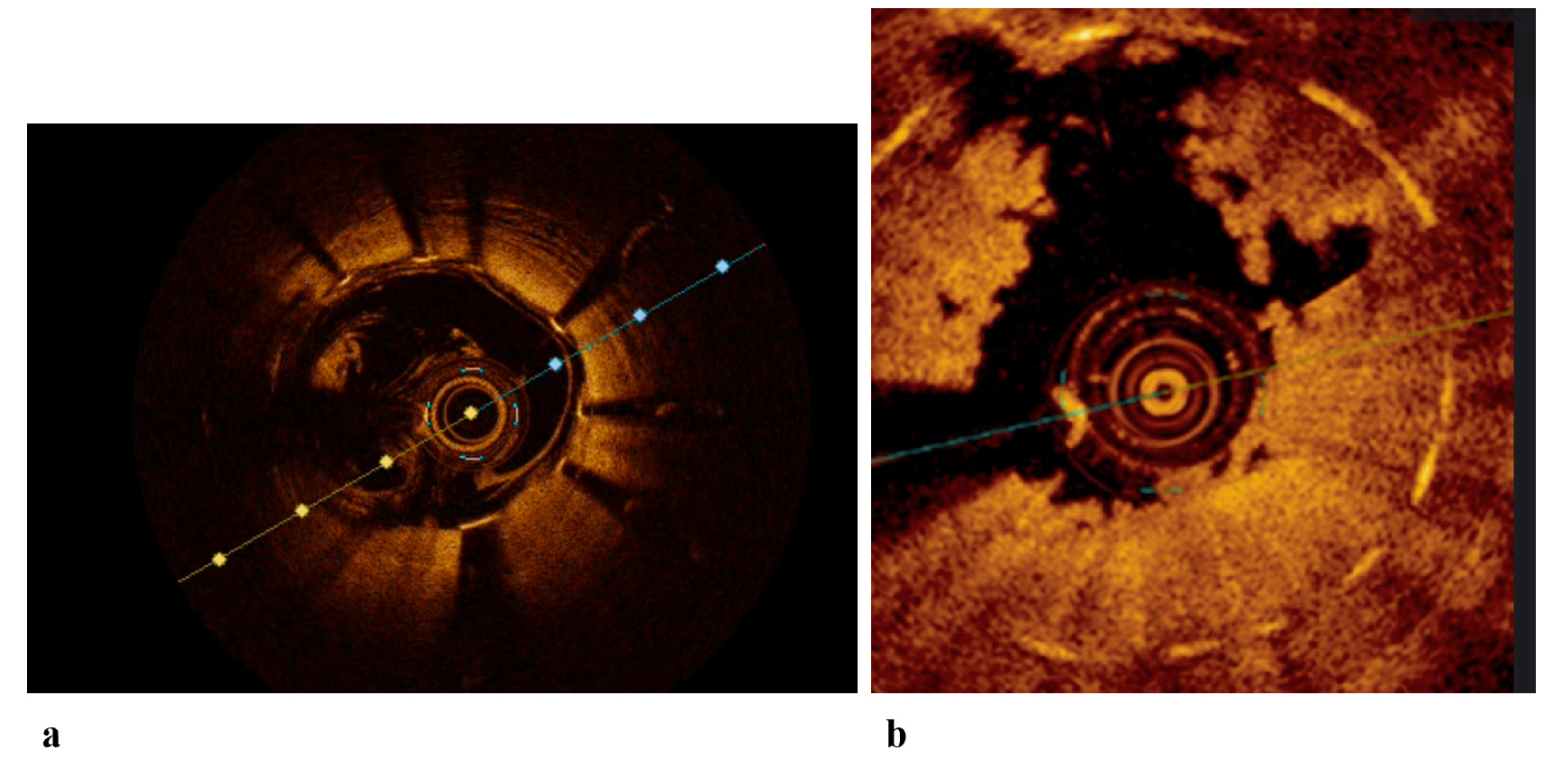
**OCT images of stented coronary arteries**. (a) OCT image of a well-expanded and well-apposed stent, with presence of blood artifact. (b) OCT image of an in-stent ACS with high thrombotic burden. ACS, Acute Coronary Syndrome; OCT, Optical Coherence Tomography.

OCT employs near-infrared light delivered to the vessel wall via a rotating single optical fiber, housed within a short monorail imaging sheath that includes an integrated imaging lens. By capturing the amplitude and time delay of backscattered light, OCT produces high-resolution, cross-sectional, and three-dimensional volumetric representations of vascular microarchitecture. Given the rapid propagation of light, interferometric techniques are essential for detecting backscattered signals. This involves splitting the light beam into reference and signal components and measuring the resulting interference pattern based on differences in their optical frequencies. Because blood strongly scatters near-infrared light and significantly attenuates the OCT signal, intraluminal flushing is required to displace blood during image acquisition (Fig. 1). The shorter wavelength of OCT’s infrared light (approximately 1.3 µm) compared to that of IVUS (~40 µm at 40 MHz) allows for superior axial resolution (10–20 µm vs. 50–150 µm). However, this comes at the expense of reduced tissue penetration (1–2 mm vs. 5–6 mm), which can limit imaging, particularly in the presence of highly attenuating materials such as red thrombus or lipid-rich/necrotic core plaques. To obtain a high-quality OCT image, it is important to have stable engagement of the coronary ostium to achieve total blood clearance during the OCT run, by adequate contrast injection. Despite contrast being the standard way to remove blood, other agents as dextran or simple saline, have been tested as a way to reduce total contrast volume [6].

## 3. OCT in ACS

**Fig. 2.**
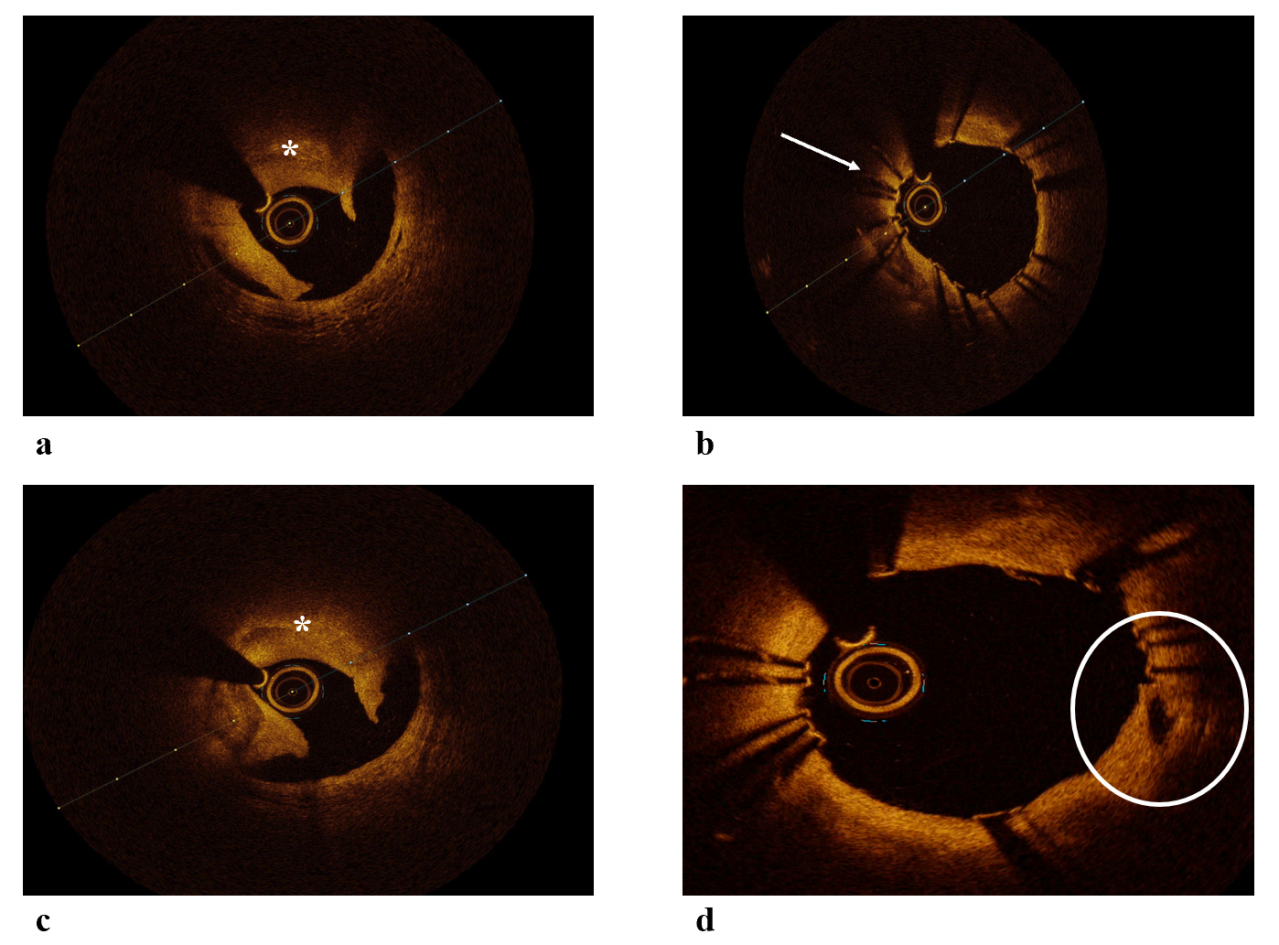
**OCT images of ACS**. (a) OCT image of plaque rupture and red thrombus leading to ACS. (b) OCT image showing a segment of stent underexpansion leading to ACS. (c) OCT image of plaque rupture and red thrombus leading to ACS. (d) OCT image showing a plaque rupture covered by stent. White arrow: underexpanded segment, Circle: plaque rupture. ACS, Acute Coronary Syndrome; OCT, Optical Coherence Tomography. *: thrombus.

A key advantage of OCT lies in its capacity to characterize plaque morphology at the site of culprit lesions in ACS. In 2013, a diagnostic algorithm was developed to differentiate underlying plaque phenotypes—including plaque rupture, plaque erosion, and calcified nodules—based on OCT imaging. This algorithm was derived from a cohort of 128 patients with ACS enrolled in the Massachusetts General Hospital OCT registry [7, 8]. This study demonstrated that OCT is capable of distinguishing among different plaque phenotypes at culprit lesion sites, revealing a distribution of plaque types that closely mirrors findings from prior histopathological investigations. Thrombus is the predominant finding of ACS, and OCT can discriminate between red and white thrombus with high accuracy. In patients with obstructive atherosclerotic coronary artery disease presenting with ACS, the most frequently encountered underlying lesion is a ruptured lipid-rich plaque [9]. By OCT, plaque rupture is characterized by a break in the fibrous cap, typically accompanied by an underlying cavity within a lipid-rich core. Thrombotic material, often overhanging the site of rupture, is commonly observed in patients with acute presentations; however, its absence does not exclude the diagnosis (Fig. 2). This is because early administration of antiplatelet and antithrombotic therapy may lead to thrombus resolution before coronary angiography, and mechanical removal may occur if aspiration thrombectomy is employed. Although plaque rupture remains the most common finding in ACS, histopathological studies have identified plaque erosion in approximately 20–30% of cases [8]. Differentiating between plaque rupture and erosion using OCT may have clinical utility in guiding treatment decisions. For instance, in cases of plaque erosion with non-critical luminal narrowing, deferral of stent implantation may be considered [10, 11].

Clinical settings as myocardial infarction with non-obstructive coronary artery disease (MINOCA), spontaneous coronary artery dissection (SCAD), and stent failure (SF) presenting as ACS are also ACS that mandate a different approach. Cardiac magnetic resonance imaging (MRI) is a key examination for the final diagnosis of MINOCA; however, OCT can depict the pathogenic source during the acute setting and guide our initial approach [12]. Angiography has significant limitations in SCAD diagnosis that may be overcomed by OCT—keeping always in mind that a powerful contrast injection can propagate a dissection [13]. SF cause should always be pursued, as it guides the index procedure and fixing it prevents future events [14]. Those subsets are analyzed further in the next section.

Coronary thrombosis following plaque rupture or erosion does not invariably lead to clinical manifestations. In some instances, plaque rupture may occur without the development of an occlusive thrombus, allowing healing to proceed via endogenous antithrombotic mechanisms. Autopsy studies have demonstrated evidence of multiple prior rupture sites beneath an acute rupture, often visualized as layered tissue structures overlying a necrotic core [15]. OCT has the capability to detect these layered patterns within coronary plaques, which correspond to sites of healed plaque and have been validated through histopathological correlation [16]. Consistent with earlier pathological findings, it is proposed that episodes of plaque disruption followed by thrombus organization contribute to accelerated lesion progression, manifesting as a layered appearance on OCT [15, 17]. Consequently, OCT-defined healed coronary plaques—characterized by their distinct layered morphology—serve as imaging markers of prior plaque instability and reparative processes.

## 4. The Role of OCT in Specific ACS Clinical Subsets

### 4.1 OCT for the Detection of Thrombus 

Thrombosis plays a central role in the pathophysiology of ACS, which is primarily triggered by damage to atherosclerotic plaques through rupture, erosion, or calcified nodules. These processes expose thrombogenic material such as tissue factor and subendothelial collagen to the bloodstream, initiating platelet activation and the coagulation cascade, ultimately leading to thrombus formation within the coronary arteries. This thrombus can partially or completely occlude the vessel lumen, impairing coronary blood flow and causing myocardial ischemia and infarction [18, 19, 20, 21]. Plaque rupture is the predominant mechanism in about 70% of ACS cases. It involves the disruption of a vulnerable plaque characterized by a thin fibrous cap and a lipid-rich necrotic core. Inflammatory mediators such as matrix metalloproteinases degrade the fibrous cap collagen, weakening the plaque’s structural integrity and causing rupture. This exposes highly thrombogenic material that triggers platelet aggregation and thrombus formation [18]. In some cases, plaque rupture occurs at sites of calcified nodules, which can also contribute to thrombosis [22]. Superficial endothelial erosion accounts for approximately 30% of ACS cases and is more common in women and diabetics with hypertriglyceridemia. It involves damage to the endothelial layer without deep plaque rupture, possibly mediated by matrix metalloproteinases disrupting endothelial cell attachment. This leads to thrombus formation on an intact fibrous cap, often resulting in non-occlusive thrombi that may heal and contribute to progressive atherosclerosis [23]. Coronary artery spasm can also contribute to thrombosis by inducing endothelial injury and promoting thrombin generation, further enhancing the prothrombotic state [23]. Elevated levels of tissue factor, primarily expressed by macrophages within plaques, initiate the extrinsic coagulation cascade and play a critical role in thrombogenicity in ACS. OCT has emerged as a pivotal tool in investigating these thrombotic mechanisms (Fig. 2). Its high-resolution imaging capabilities allow detailed visualization of intraluminal and vessel wall structures, enabling detection and differentiation of thrombus types—red thrombi (rich in red blood cells) versus white thrombi (platelet-rich)—and identification of non-occlusive thrombi that may be missed by other imaging modalities. OCT also facilitates the assessment of plaque morphology, including ruptures, erosions, calcified nodules, and SCAD, thereby improving diagnostic accuracy and guiding personalized treatment strategies in ACS [24, 25]. Furthermore, the pathogenesis of ACS involves a complex interplay of inflammatory and immune processes. Activated T cells, particularly CD4+CD28 T cells, infiltrate unstable plaques and release proinflammatory cytokines like interferon-gamma, which activate macrophages and induce apoptosis of vascular smooth muscle cells. This weakens the fibrous cap and promotes plaque instability and rupture, linking adaptive immunity to thrombotic events in ACS [19].

### 4.2 OCT for the Detection of Plaque Rupture

Plaque rupture is the most frequent finding in patients with ACS and is responsible for 65% of ACS [26]. It is associated with worse outcomes after angioplasty and more commonly presents with the ST-segment elevation myocardial infarction phenotype [27]. Plaque rupture commonly arises in the setting of OCT-defined thin-cap fibroatheromas (TCFA) and is characterized by morphological features such as intimal tearing, fibrous cap disruption, or dissection (Fig. 2). When the vessel is flushed with optically transparent crystalloids or radiocontrast agents, these structural defects may exhibit minimal or absent OCT signal, often appearing as intraplaque cavities. The distinction between plaque rupture and erosion has important therapeutic implications and may inform treatment strategies, including the potential to defer stent implantation in cases of plaque erosion associated with non-critical stenosis. Due to its higher spatial resolution, OCT is superior in detecting plaque rupture that IVUS [28]. Also, research based on OCT imaging has shown that the plaque rupture phenotype ACS is associated with worse outcomes that intact fibroatheroma [29]. Toutouzas *et al*. [30] demonstrated that in patients with ACS, culprit ruptured plaques are not evenly distributed throughout the coronary vasculature but are more frequently localized to the proximal segments of the coronary arteries.

### 4.3 OCT for the Detection of Plaque Erosion

The defining histopathological feature of plaque erosion is the absence of endothelial lining over the affected plaque and the prevalence in ACS is about 25% to 30% [26]. Although OCT offers excellent spatial resolution, it lacks the capacity to directly visualize endothelial integrity. As a result, the diagnosis of plaque erosion using OCT is primarily made by excluding the presence of fibrous cap rupture [8]. Characteristic OCT findings suggestive of plaque erosion include the presence of white thrombus overlying an intact fibrous cap, an irregular luminal surface without associated thrombus or thrombus obscuring the underlying plaque in the absence of adjacent lipid-rich plaque or calcification proximal or distal to the thrombotic site. Plaque erosion is considered “definite” when a thrombus is identified overlying an intact fibrous cap. In contrast, it is deemed “probable” in the absence of both thrombus and fibrous cap rupture, provided that irregularities of the luminal surface are present. The EROSION trials tried to investigate and create a new therapeutic landscape for ACS due to plaque erosion, offering the chance of conservative treatment [11, 27]. Double antiplatelet treatment instead of stent implantations was safe when plaque erosion resulting in stenosis <70% was identified by OCT [10]. The OCT analysis as well as the angiography, were improved with less erosion and less thrombotic material at 1-year follow-up [27]. In the randomized EROSION III trial, the use of OCT in patients with ST-segment elevation myocardial infarction and early infarct-related artery patency was associated with a reduced frequency of stent implantation during primary percutaneous coronary intervention, compared to angiographic guidance alone [11].

### 4.4 OCT for the Detection of Calcified Nodules 

Calcified coronary artery lesions, particularly calcified nodules (CNs), are the least common but critical contributors to ACS and are accountable for 2% to 7% of them [31]. These complex structures drive luminal narrowing through a unique pathophysiology involving chronic inflammation, osteogenic transformation, and extracellular matrix remodeling [32, 33]. Intravascular imaging, especially OCT, has become indispensable for diagnosing CNs and guiding PCI. CNs can arise from a combination of mechanical stress and biological processes. Chronic Inflammation plays an important role with macrophages and T-cells infiltrate the plaque, releasing cytokines (e.g., IL-6, TNF-α) that promote calcification and destabilization of the fibrous cap [32]. Moreover, osteogenic transformation in terms of the phenotypic change of vascular smooth muscle cells, expressing bone-forming proteins like osteocalcin and alkaline phosphatase, leading to microcalcifications, has a crucial role on the development of CN, together with the degradation of collagen that weakens the plaque structure and increases risk of rupture [32, 34, 35]. CNs are classified as eruptive (associated with thrombus formation and acute rupture) or non-eruptive (stable, calcified protrusions), each requiring distinct management strategies [36]. OCT allows for the visualization of fibrous cap disruption, protruding calcifications, external plaques, and calcifications both proximal and distal to the plaque. TCFA (<65 µm) overlying CNs are linked to plaque vulnerability [37], while OCT can also differentiate protruding calcifications as irregular, superficial calcium deposits with sharp edges, often causing lumen compromise and also differentiate eruptive vs. non-eruptive CNs [38].

### 4.5 The Role of OCT in the MINOCA

MINOCA represents a syndrome of various unknown causes, characterized by symptoms typical of myocardial infarction and the presence of myocardial necrosis, without occlusion of large subepicardial coronary arteries. MINOCA is classified according to its underlying causes as either atherosclerotic or non-atherosclerotic. In MINOCA, microcirculatory dysfunction is most often caused by a non-atherosclerotic mechanism, which serves as the leading pathophysiological factor [39]. Non-Atherosclerotic Causes of MINOCA include coronary microvascular dysfunction, coronary artery spasm, spontaneous coronary artery dissection, supply–Demand mismatch (Type II MI), and thromboembolism from distant causes. OCT plays a vital role in diagnosing and managing myocardial infarction with MINOCA, a condition where patients have heart attack symptoms but no significant coronary artery stenosis on angiography. OCT’s high-resolution imaging allows detailed visualization of the coronary artery wall, uncovering subtle abnormalities that angiography alone often misses.

Studies have shown that OCT can identify various underlying causes of MINOCA, such as plaque rupture, plaque erosion, calcified nodules, thrombus formation, and spontaneous coronary artery dissection (SCAD). For example, in one prospective study, OCT detected atherosclerotic causes in about 36% of MINOCA patients, leading to changes in treatment plans including percutaneous coronary intervention or modification of antithrombotic therapy in some cases [40], while another study found that combining OCT with cardiac magnetic resonance imaging (CMR) provided a diagnosis in 100% of MINOCA patients, highlighting the complementary nature of these tools—OCT excels at detecting coronary artery pathology, while CMR identifies myocardial injury [41]. OCT helps differentiate between atherosclerotic and non-atherosclerotic mechanisms, which is crucial because treatment strategies differ. Patients with atherosclerotic lesions detected by OCT may benefit from statins and ACE inhibitors, which reduce adverse cardiac events, whereas routine dual antiplatelet therapy may not be universally effective in MINOCA. Furthermore, OCT findings have prognostic value; patients with high-risk lesions identified by OCT tend to have worse outcomes and may require closer follow-up and tailored therapy [42]. Early use of OCT can detect hyperacute features such as thrombus or plaque disruption that might be missed if imaging is delayed, ensuring timely and accurate diagnosis. This precision in identifying the culprit lesion helps guide personalized treatment strategies, improving prognosis and reducing the risk of recurrent events [43].

### 4.6 Spontaneous Coronary Artery Dissection 

SCAD is characterized by the entry of blood into the layers of the coronary artery wall, leading to the formation of a false lumen that compresses the true lumen. This compression impairs coronary blood flow and precipitates ACS. The pathophysiology involves either an intimal tear allowing blood from the lumen to enter the vessel wall (“inside-out” hypothesis) or bleeding from the vasa vasorum causing an intramural hematoma without intimal rupture (“outside-in” hypothesis) [44]. Both mechanisms result in the separation of arterial wall layers and the formation of an intramural hematoma that compresses the true lumen, causing myocardial ischemia. SCAD is notably one of the most common causes of ACS in young individuals, especially young women, frequently occurring in peripartum periods and associated with conditions such as fibromuscular dysplasia. Unlike atherosclerotic disease, SCAD typically occurs without lipid-filled plaques or significant vessel wall inflammation. OCT plays a crucial role in diagnosing SCAD, particularly when angiographic findings are inconclusive. OCT enables detailed visualization of key features such as intramural hematomas, intimal tears, intima-medial flaps, and older scarred dissections. It can detect the presence or absence of fenestrations between true and false lumens, which influences the pressure dynamics within the vessel wall and guides clinical management. This imaging precision helps determine the appropriate treatment strategy, including the decision to pursue PCI or conservative management [45].

### 4.7 The Role of OCT in Stent Failure

**Fig. 3.**
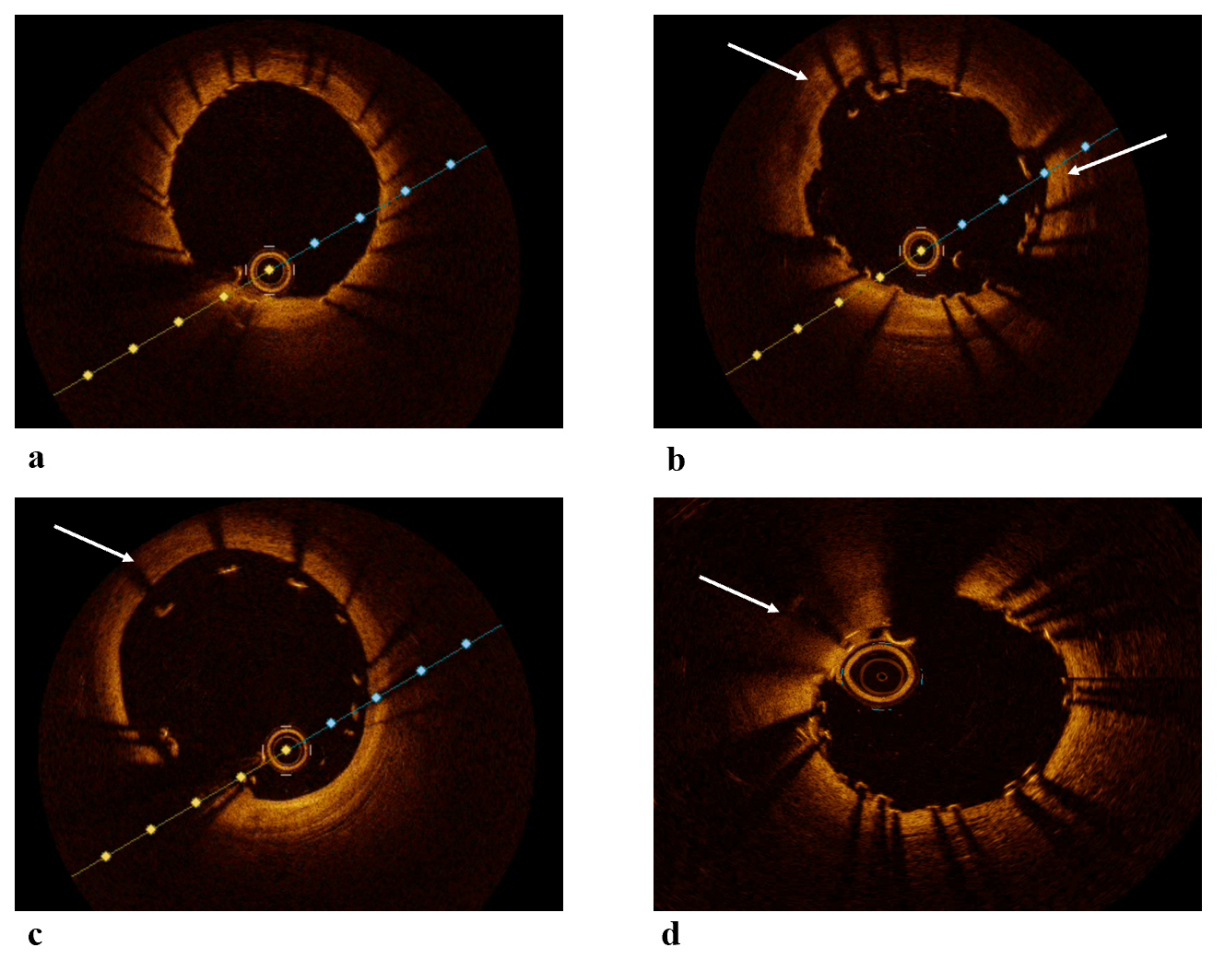
**OCT images post-PCI**. (a) OCT image showing a well-expanded and well-apposed stent. (b) OCT image showing malapposition of the stent. (c) OCT image showing significant malapposition of the stent. (d) OCT image showing a segment of stent underexpansion. White arrows: malapposed segment in figures b,c and underexpanded segment in image d. OCT, Optical Coherence Tomography; PCI, Percutaneous Coronary Intervention.

Despite advancements in PCI devices, pharmacological treatments, and procedural techniques, the occurrence of SF remains a significant concern [46]. SF can manifest either as stable angina or myocardial infarction (MI), and it may involve in-stent restenosis (ISR) or stent thrombosis (ST). Research utilizing OCT has demonstrated its capability to detect various causes of SF, such as uncovered stent struts, malapposition, and excessive tissue growth (Figs. 1,3). Through detailed imaging analysis, the composition of tissue within the stent can be classified into lipid-rich, fibrotic, or calcified types. Additionally, imaging techniques can reveal issues like stent underexpansion and the presence of thrombus. OCT’s high-resolution imaging allows detailed assessment of stent apposition, expansion, and vessel wall morphology, enabling identification of key mechanisms contributing to SF, such as uncovered stent struts, malapposition, underexpansion, and thrombus formation. Importantly, OCT provides precise characterization of neointimal tissue composition, differentiating lipid-rich, fibrotic, or calcified tissue, which aids in understanding restenosis mechanisms and tailoring treatment strategies. The presence of neoatherosclerosis or multiple stent layers on OCT has been associated with increased risk of recurrent SF, highlighting the need for optimized intervention. Quantitative OCT parameters, particularly minimal stent area (MSA) and stent expansion relative to reference vessel size, have been strongly linked to clinical outcomes. The ILUMIEN IV trial, a large prospective study, demonstrated that smaller MSA and proximal edge dissections independently predict target lesion failure, cardiac death, and stent thrombosis at two years post-PCI [47]. Similarly, the CLI-OPCI II registry confirmed that suboptimal stent implantation—defined by OCT criteria including MSA <4.5 mm^2^, edge dissections, and residual reference segment disease—is common and significantly associated with major adverse cardiac events (MACE) [48, 49]. OCT’s ability to detect thrombus and subtle edge dissections also informs intraprocedural decision-making, guiding additional interventions such as balloon post-dilation or further stenting to optimize stent deployment and reduce adverse events [4, 46]. These findings underscore the critical role of OCT-guided PCI in improving procedural outcomes and long-term prognosis in patients undergoing stent implantation.

### 4.8 OCT and Neoatheromatosis

OCT has become a vital tool for identifying neoatherosclerosis, which is increasingly recognized as an important cause of very late SF. Thanks to its exceptional resolution, OCT can clearly visualize features such as lipid-rich plaques, thin fibrous caps, macrophage accumulation, and calcifications within the neointima that develop inside stents over time. These detailed images help distinguish neoatherosclerosis from other forms of in-stent restenosis or thrombosis, which can be challenging with angiography or IVUS alone. OCT also reveals subtle signs like healed plaque ruptures and layered neointimal tissue, shedding light on the ongoing processes of plaque destabilization and healing that contribute to late stent complications. This level of insight is crucial for accurately diagnosing the cause of SF and guiding clinical decisions [37]. Beyond diagnosis, OCT plays a key role in guiding treatment strategies for neoatherosclerosis. By providing a precise assessment of lesion morphology and extent, OCT helps interventional cardiologists tailor their approach, whether that involves balloon angioplasty, drug-coated balloon therapy, or placing additional stents. It also allows for careful evaluation of stent expansion and apposition, addressing mechanical factors that may promote disease progression. Furthermore, OCT can detect thrombus and subtle dissections around the lesion, enabling timely interventions to reduce the risk of recurrent events. Detailed tissue characterization also supports decisions about medical therapy, such as intensifying antiplatelet or lipid-lowering treatments based on plaque vulnerability. Overall, OCT-guided management has been shown to improve procedural outcomes and may help prevent very late SF related to neoatherosclerosis [50, 51, 52].

### 4.9 OCT for the Recognition of the Vulnerable Plaque

Predicting which atherosclerotic plaques will trigger clinical instability and become the culprit lesions in ACS has long been a significant challenge in cardiovascular medicine. A vulnerable or unstable plaque may often involve the formation of thrombus within the plaque or vessel lumen; however, they do not always result in angina or complete vessel obstruction [53]. As a result, these lesions can remain asymptomatic for extended periods until one of these cycles culminates in a clinical event, frequently presenting as myocardial infarction or sudden death [54]. In retrospective analyses, interventional cardiologists and cardiovascular pathologists commonly designate the lesion causing coronary occlusion and subsequent death as the ‘culprit plaque’, independent of its underlying histopathologic characteristics. For prospective clinical assessment, however, an analogous terminology is required to characterize plaques that may precipitate future adverse events [55]. Coronary vulnerable plaques are typically marked by a thin fibrous cap covering a large lipid-rich necrotic core, which makes them susceptible to rupture and subsequent thrombotic events. These plaques often exhibit active inflammation, characterized by macrophage infiltration, and demonstrate expansive or positive remodeling of the vessel wall, where the artery enlarges outward to accommodate plaque growth without significantly narrowing the lumen. Additional features include spotty calcifications, neovascularization within the plaque (vasa vasorum proliferation), and intraplaque hemorrhage, all of which contribute to plaque instability. The combination of these structural and biological factors creates a high mechanical stress environment on the fibrous cap, increasing the likelihood of rupture and ACS such as myocardial infarction [56, 57, 58]. Unlike stable plaques, which tend to have thick fibrous caps and smaller lipid cores, causing gradual luminal narrowing and stable symptoms, vulnerable plaques often maintain a relatively preserved lumen until rupture occurs.

OCT is uniquely suited to detect coronary vulnerable plaques due to its high-resolution imaging capability, which allows detailed visualization of plaque microstructures *in vivo*. OCT identifies key features of vulnerable plaques, including TCFAs characterized by a fibrous cap thickness less than 65 micrometers overlaying a large lipid-rich necrotic core. It can also detect macrophage accumulation near the fibrous cap, which indicates active inflammation, plaque fissures as well as intraplaque microvessels, cholesterol crystals, and areas of intraplaque hemorrhage. These features are strongly associated with plaque instability and a higher risk of rupture, leading to ACS [59]. OCT’s ability to precisely measure fibrous cap thickness and differentiate tissue types with high sensitivity and specificity makes it a powerful tool for identifying plaques at high risk of causing clinical events

Beyond structural assessment, OCT can characterize plaque composition and detect thrombus, calcified nodules, and healed plaque ruptures, providing insights into the dynamic processes of plaque progression and healing. Studies have shown that non-culprit plaques identified by OCT as both lipid-rich and thin-capped are significantly associated with future ACS events, highlighting OCT’s prognostic value [60, 61]. Furthermore, advances in automated image analysis, including AI-based algorithms, are enhancing OCT’s diagnostic accuracy and helping clinicians identify patients with vulnerable plaques who may benefit from intensified medical therapy or closer monitoring [62, 63]. Histological analysis suggest that macrophage infiltration diversity and calcification characteristics may reveal plaque vulnerability. Severe sheet calcification is a marker of stable plaque, whereas fragmentation or microcalcification are markers of unstable plaques. OCT catheters combined with IVUS and infrared spectroscopy may provide ways to translate more histological patterns to intravascular images [64].

## 5. OCT Disadvantages

Despite being a valuable asset, OCT is costly and carries certain disadvantages. OCT run requires a vessel free of blood; contrast is the standard way for blood clearance increasing the risks of iodine contrast agents. Because optimal coronary preparation is essential, residual blood pooling produces high-intensity signals that degrade and distort the final OCT image. That is, also, the reason that OCT is not the preferred modality for aorto-ostial lesions and left main lesions. A power contrast injection carries the risk of intimal trauma and dissection. That is of particular importance in ACS and proximal SCAD, as the injection may propagate trauma and intramural hematoma [65]. The main limitation of OCT analysis is the tissue penetration depth. Current maximum tissue penetration is only 1.5–3 mm making it challenging to fully characterize an atheromatous plaque. OCT provides precise delineation of the lumen–vessel wall interface; however, its limited depth of penetration restricts visualization of the entire vessel architecture when compared with IVUS. Far-field detection is limited with OCT [66]. As the guidewire does not extend along the full length of the OCT catheter, its silhouette consistently appears within the images, resulting in localized reductions in image quality.

## 6. Conclusion

OCT is a valuable tool in the diagnosis and management of ACS. Despite certain limitations, OCT enables detailed characterization of ACS subtypes and facilitates procedural optimization, potentially improving long-term clinical outcomes. When compared with IVUS, neither modality can be deemed superior, as each possesses distinct advantages and inherent constraints. Ongoing investigations are exploring the integration of both imaging techniques within a single catheter to harness their complementary strengths.

## Abbreviations

ACS, acute coronary syndrome; CCTA, Coronary Computed Tomography Angiography; CN, calcified nodule; IVUS, intravascular ultrasound; MACE, major adverse cardiac events; MRI, magnetic resonance imaging; MSA, minimal stent area; MINOCA, myocardial infarction with non-obstructive coronary artery disease; OCT, optical coherence tomography; PCI, percutaneous coronary intervention; SCAD, spontaneous coronary artery dissection; TCFA, thin-cap fibroatheromas; TD-OCT, time-domain detection technology.
